# Improved cholera control in Kenya: A retrospective analysis of 2017–2019 in Nairobi and Homabay

**DOI:** 10.4102/jphia.v15i1.741

**Published:** 2024-11-22

**Authors:** Kyeng Mercy, Ganesh Pokhariyal, Noah T. Fongwen, Nicaise Ndembi, Lucy Kivuti-Bitok

**Affiliations:** 1 Department of Medical Microbiology, Faculty of Health Sciences, University of Nairobi, Nairobi, Kenya; 2 Department of Surveillance and Disease Intelligence, Africa Centres for Disease Control and Prevention, Addis Ababa, Ethiopia; 3 Department of Mathematics, University of Nairobi, Kenya; 4 Department of Mathematics, Graphic Era Hill University, Dehradun, India; 5 Department of Laboratory Networks and Systems, Africa Centres for Disease Control and Prevention, Addis Ababa, Ethiopia; 6 Department of Nursing, Faculty of Health Sciences, University of Nairobi, Nairobi, Kenya

**Keywords:** Kenya, cholera, surveillance, response, evaluation

## Abstract

**Background:**

Kenya has recorded at least 38 678 cases and 695 deaths over the last decade, and costing on average $2.2 million annually. From 2014 to 2016, the country experienced one of the deadliest and largest outbreak. However, between 2017 and 2020, there was a decline in the number of reported cases and deaths.

**Aim:**

This study seeks to reveal the investments made post the 2014–2016 outbreak and highlight existing gaps that need to be addressed to stop the resurgence of cholera outbreaks in Kenya.

**Setting:**

The study was conducted in two counties: Homabay and Nairobi.

**Methods:**

We used an observational study. Data were collected from 20 health facilities (involved in cholera control, during the study), 9 key informant interviews (KII) and 6 focus group discussions (FGDs).

**Results:**

We found improvement such as: dissemination of standard operating procedures, aligned reporting system, field epidemiology programme, establishment of a public health emergency operating centre and improved partner coordination. On the other hand, 12 of the selected 20 (60%) facilities had no prior training before government financing and laboratory capacity was sub-optimal: 13 (65%) facilities had no prior training, 16 (20%) had no operational laboratory plan and 10 (50%) had inadequate laboratory test kits and reagents.

**Conclusion:**

This study highlights that Kenya has experienced an improvement in specific core capacities.

**Contribution:**

For Kenya to completely flatten the curve, there is need for more sustainable investment and government’s commitment in health system strengthening.

## Introduction

**FIGURE 1:**
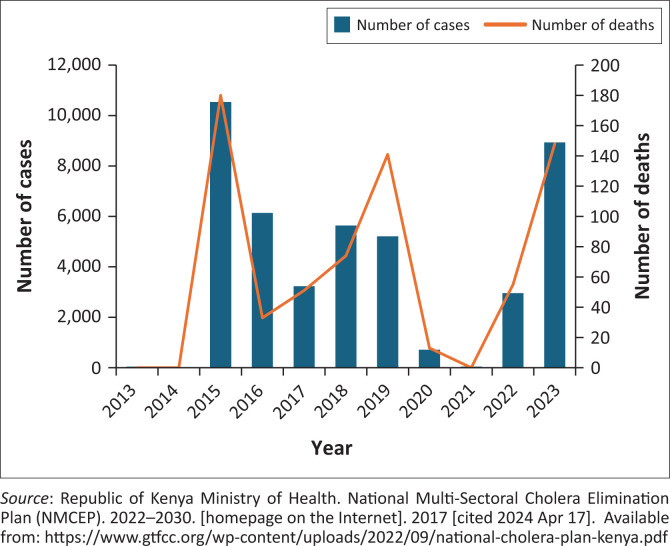
Trend of cholera cases in Kenya from 2013 to 2023.

In the last 10 years, the world has seen a global rise in the number of cholera cases reported. Cholera continues to pose a significant threat to Kenya’s national health system and has caused at least 38 678 cases (including confirmed and clinically compatible cases) and 695 deaths over the last decade.^1^ Within Africa, Kenya has experienced frequent cholera outbreaks, with more than 15 discrete outbreaks from 1971 to 2010. In 2015, Kenya accounted for 19% of the 172 454 reported cases and 7% of the 1304 globally reported deaths.^2^ During 2014–2016, Kenya experienced one of the deadliest and largest cholera outbreaks since 2010, with at least 16 840 cases and 256 deaths reported from 30 of the 47 counties. This outbreak had far-reaching impacts on the quality of life and the country’s economy costing over $2.2m annually.^3^ Since then, the country has been reporting cholera cases almost annually in certain high-risk areas. Factors such as open defecation, high population density in urban slums, increased cross-border movement of persons from countries reporting cholera outbreaks amid humanitarian crises and the growing climate change with increased incidence of flooding were some of the key drivers of the outbreaks reported in Kenya.^1^ The Global Task Force on Cholera Control launched an initiative to eradicate cholera by 2030 and developed a roadmap that focusses on: prompt detection and effective response; a multi-sectoral approach to detect cholera resurgence in the endemic areas; a productive partnership mechanism of coordination for surveillance, research, and monitoring and evaluation, resource mobilisation and advocacy. Aligning with this vision, the Kenyan government has targeted to reduce the cholera annual incidence to 0 per 100 000 by 2027 and have prioritised the administration of oral cholera vaccine (OCV) to identified hotspots to curb the spread of the disease.^2,4^ Between 2017 and 2019, Kenya recorded a 16% decrease in cases compared to the 2014–2016 outbreak (Figure 1).^4^ This study seeks to understand the improvements made in the surveillance and response capacity post-2014–2016 that led to improvements in containing the outbreak despite health system challenges and competing health emergencies such as climate change.

This evaluation focussed on the assessment of the ability of Kenya’s health system to detect and respond promptly to cholera cases during the 2017–2019 outbreak. The study builds on previous cholera capacity evaluations conducted in 2016, and aims to identify the current state of cholera investments in Kenya and assess the existing gaps in prevention and control efforts to mitigate the risk of future outbreaks.

## Research methods and design

This observational study employed a mixed method of qualitative and quantitative data collection to evaluate cholera surveillance and response data at the county, facility and community levels. The quantitative and qualitative datasets were given equal weight in analysis and comparison. The study was a non-probability purposive sampling carried out in four sub-counties in Nairobi (Kibra, Embakasi, Dagoretti and Kasarani) and two in Homabay (Homabay town and Ndhiwa).

### Study participants

Participants for the key informant interviews (KII) included health facility surveillance focal points (Integrated Disease Surveillance and Response Strategy [IDSR] focal point) or hospital officer in charge (where there is not a disease surveillance focal point) and district medical officers. A total of nine key informants were interviewed (four females and five males). Participants for the focus group discussions (FGD) included community health workers (community extension workers) and public health officers. Only those facilities involved in cholera surveillance within the study area (Dagoretti, Kibra, Embakasi, Kasarani, Ndhiwa and Homabay Town) were included in the health facility assessment.

#### Sampling procedure and justification

Health facilities were selected based on the following criteria: they were the first point of referral for all suspected cholera cases, they had a laboratory capacity to carry out cholera diagnosis and finally they had reported cases of both suspected and confirmed cholera for 2014–2016 and 2017–2019 cholera outbreaks in the Kenya health information system.^5^ A total of 20 healthcare facilities and 36 community healthcare workers were recruited from the sub-counties.

### Data collection

Data on Kenya’s capacity to detect and respond to the 2017–2019 cholera outbreak were collected from the 20 health facilities using a questionnaire developed from three guidelines: IDSR, the updated guideline for evaluating surveillance systems and the Africa Centres for Disease Event-based Surveillance (EBS) framework. Nine KIIs and six FGDs were conducted to gain more insights on the capacity of Kenya’s surveillance system. Capacity areas were assessed using a consolidation of surveillance variables of three key pillars: system resources, system structures and core functions anchored on good governance and leadership.^6^

### Data analysis

The data captured using questionnaires were programmed into Comcare software and exported to Stata 5 (STATA Corp [2017] STATA Statistical Software: Release 15. College Station, Texas, United States [US]: StataCorp LLC) for error checking and cleaning. Data from reviewed forms were additionally keyed in Microsoft Excel and exported to STATA for cleaning before analysis. All FGDs and KII were audio-recorded and transcribed into English. Transcripts were reviewed and a codebook was generated.

### Ethical considerations

Ethical approval to conduct this study was obtained from the Kenyatta National Hospital University of Nairobi Ethics Review Committee (No. KNH/ERC/R/71). Written consent was also obtained from all the research participants.

## Results

During the 2017–2019 outbreak, a total of 14 074 cholera cases and 266 deaths (case fatality rate [CFR]: 1.9%) were reported from 20 counties in Kenya. This represented a 16% decrease in the number of cases compared to the 2014–2016 outbreak where at least 16 500 cases were reported.^7^ During the 2017–2019 outbreak, a total of 333 cholera cases and 9 deaths (CFR: 2.7%) from cholera were reported from the two sampled counties^1,5,8^: Nairobi (258 cases; 7 deaths) and Homabay (75 cases; 2 deaths). This represents an 85% decrease in the number of cases reported compared to the 2014–2015 period in these counties.^1,5,8^ Table 1 provides an analysis of capacities across core areas, examining changes between the periods 2014–2016 and 2017–2019.

**TABLE 1:** Analysis of capacities across core areas, examining changes between the periods 2014–2016 and 2017–2019.

Capacity	2014–2016	2017–2019
RRT	Not deployed.	Deployment of first batch of FELTP to national and sub-national.
Cholera preparedness and response plan	Not in place at the beginning of the outbreak.	Was in place to guide the response.
Funding	Minimal government support, majority was doner support. Onset of devolved counties.	Minimal partner support and reduced government investment. Counties had better adapted to the devolution agenda.
Legislation, guidelines and SOPs	Second edition of IDSR implemented in < 20% of facilities. Case definitions, SOPs available in < 20% of facilities.	All sub-counties had implemented IDSR strategy. Ninety-one per cent had case definitions.
Emergency operating centre	Not activated.	Was in place and activated for the first time.

IDSR, Integrated Disease Surveillance and Response; RRT, Rapid Response Team; SOPs, standard operating procedures; FELTP, Field Epidemiology and Laboratory Training Program.

### Surveillance governance

#### Placement of Kenya’s National Cholera Surveillance Unit and structure

During the 2017–2019 cholera outbreak, there was no functional national public health institute in place.^9^ However, the Government of Kenya had established the Division of Disease Surveillance and Response (DDSR) to build an effective, efficient public health surveillance and response system. At the regional level (County and sub-County), 70% of the responders indicated the presence of a trained Rapid Response Team (RRT), and Disaster Preparedness and Management Committees in charge of disease preparedness and response. In the health facilities, 60% had RRT and surveillance unit for monitoring, assessing and responding to cholera outbreaks.

#### Health financing

Kenya just like majority of the African countries had not met its target of allocating at least 15% of government’s total budget in healthcare during both the evaluation periods. All African Union Member States pledged to this target of 15%, now termed the ‘Abuja declaration’.^10^ In 2013, the devolution policy in Kenya went into full force with the counties given the autonomy to mobilise and manage their resources. In the fiscal year 2013–2014, the county’s share of the total health budget was 54%. In the 2017–2018 fiscal year, their share increased to 63%, but decreased to 57% in 2018–2019.^11,12^ This decrease had severe setbacks in the management of health emergencies at country level:

‘There were challenges and some of which needed financing, and you know that most of our activities, especially in health, I’m sorry to say, is majorly financed by partners. Sometimes, we also needed support supervision, and we still depended on them because of the transport challenges.’ (FGD E, sub-county surveillance officer, male)

#### Legislations, policy guidelines and standard operating procedures

During both periods, legislations and policies that govern disease surveillance and control including cholera were in place (Appendix 1). However, the level of implementation differed across both periods. The second edition of the IDSR by the World Health Organization (WHO) was in place prior to the 2014–2016 outbreak.^13^ Cholera was listed within this strategy as one of the priority diseases in the country. Before the onset of 2014–2016, less than 20% of facilities were actively implementing the IDSR. However, by 2017 all (100%) sub-counties had case definitions and 91% had standard operating procedures (SOPs) and guidelines in place. At the facility level, while 80% had a list of priority diseases, a standard case definition of cholera and guidelines on cholera waste management, 50% (*n* = 10/20) did not have SOPs for the coordination of communication across multiple sectors and stakeholders.

Event-based surveillance had not been fully entrenched into the surveillance structure and system then:

‘No. Actually, EBS is a new concept to us. It has been implemented in other areas such as Nakuru and Mombasa but in Nairobi, only a few of us have been sensitized on it; me and a few staff from Mbagathi and Mama Lucy hospitals.’ (KII K, sub-county county surveillance officer, male)

### Resources

#### Trained workforce capacity

On trained workforce, for 2014–2016, there were no training needs assessment performed, and no staff had received any trainings on EBS. This performance was similar in 2017–2019 as 13 out of the selected 20 (65)% facilities had no training needs assessment conducted before the outbreak, 12 (60%) reported that there were no prior trainings of hospital health staff and community health promoters or supervisors on EBS and 11 (55%) indicated that there was no prior active engagement, sensitising and training of community leaders, health volunteers and other community members as appropriate, in the detection and reporting of cholera (Table 2):

‘Training needs are done after we have had an outbreak. Staffs are just donated from different departments.’ (KII W, sub-county surveillance officer, female)

‘In Kenya, training is not done as part of preparedness. Training is done after the outbreak has occurred; it is done during, not before.’ (KII D, health facility in-charge, female)

**TABLE 2:** Training information on cholera surveillance and response in Kenya.

Question	Yes (%)	No (%)	Don’t know (%)
Had staff training needs assessment been carried out in this health facility in the last year before the 2017–2019 cholera epidemic?	25	65	10
Was there a staff Training Plan in place in this facility before the cholera outbreak of 2017–2019?	50	45	5
Had the health workers in this facility been trained on how to handle and/or manage choleraoutbreaks in the last year before the cholera outbreak of 2017–2019?	35	65	0
Were there continuous, short-, or medium-term courses on cholera outbreaks or epidemiology organised in this facility before the 2017–2019 cholera outbreak?	20	70	10
Were the hospital staff trained on Event-Based Surveillance prior to or during the outbreak?	35	60	5
Were the community health volunteers/supervisors trained on EBS prior to or during the outbreak?	30	60	10
Was there an active engagement, sensitising, and training of community leaders, health volunteers, and other community members as appropriate, in the detection and reporting of cholera outbreak information before the 2017–2019 cholera outbreak?	45	55	0

**Average**	**34**	**60**	**6**

EBS, event-based surveillance.

On average, confidence in skills for cholera surveillance and response stood at 34%, significantly lower than the gold standard of 80%.

#### Non-medical countermeasures

**FIGURE 2:**
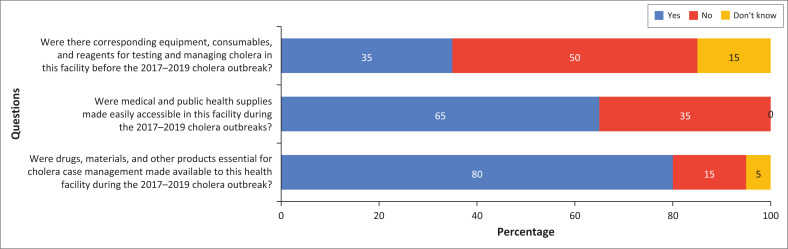
Account of resource availability.

During the 2014–2016 outbreak, non-medical supplies scored between 60% and 100%. During the 2017–2019 period, 16 out of 20 (80%) had adequate drugs, materials and other products essential for cholera case management, while 10 out of 20 (50%) indicated that consumables such as laboratory testing reagents, personal protective equipment such as gloves, masks and gowns were not available and accessible by healthcare workers. On average, the resource availability stood at 60% of the requirements (Figure 2):

‘For us who were here, that is why we are saying that the PPEs and some equipment at the community level support World Vision. The support from partners was coordinated by counties.’ (FGD D, sub-county surveillance officer, male)

#### Laboratory capacity

Less than 20% of facilities had guidelines for laboratory diagnoses prior to the 2014–2016 outbreak and furthermore the roles and responsibilities were not clear. Prior to the 2017–2019 outbreak, all the facilities had a guideline defining the roles and responsibilities, a list of reference laboratories in the country and an SOP on diagnostic testing. However, only 7 out of the 20 (35%) facilities had refresher and/or on-the-job training for laboratory staff before the outbreak and only 4 (20%) had an operational laboratory plan in place. None of the specified resources reached the 80% mark of adequacy. Specimen and material transport scored 40%, laboratory space 35%, equipment and staffing 25%, and reagents and consumables 15% (Table 3).

**TABLE 3:** Laboratory capacity.

Question	Yes (%)	No (%)	Don’t know (%)
Does a document that defines the roles and responsibilities of laboratories available at this level?	100	0	0
Was there an official list of designated national public or private reference laboratories for confirming cholera cases from this health facility before 2017–2019 cholera outbreak? Please ask to see the list.	100	0	0
Are diagnostic tests and methods used in this health facility appropriate for the laboratories as defined by national standards?	95	5	0
Were diagnostic tests and methods at this facility based on a list of priority public health risks such as cholera outbreaks before the 2017–2019 cholera outbreak?	65	30	5
Was there laboratory networking with this health facility before the 2017–2019 cholera outbreak?	65	30	5
Were laboratory resources allocated to this health facility before the 2017–2019 cholera outbreak made based on national minimal requirements?	50	45	5
Were there corresponding equipment, consumables, and reagents for testing and managing cholera in this facility before the 2017–2019 cholera outbreak?	35	50	15
Was there a plan in place for continuing education or training of laboratory staff in this facility for the last 1 year before the 2017–2019 cholera outbreak?	35	60	5
Had an inventory of laboratory capacity been carried out for laboratory materials in the last year at this level before the 2017–2019 cholera outbreak?	40	40	20
Was there an operational plan in place for strengthening laboratory services at this health facility for the last year before the 2017–2019 cholera outbreak?	25	60	15

**Average**	**61**	**32**	**7**

Challenges with the availability of Rapid Diagnostic Test (RDT) kits were reported during the 2017–2019 outbreak similar to the challenges encountered during the 2014–2016 period:

‘The issue with rapid diagnostic test kits was that they were never enough. This is a big problem. For example, we are dealing with the outbreak today, but we last tested a case several months ago.’ (KII K, health facility in-charge, female)

Unlike the RDT, the culture test takes 72 h (3 days) or up to about 2 weeks and was used to confirm the disease.

### Mechanisms and system structures

**FIGURE 3:**
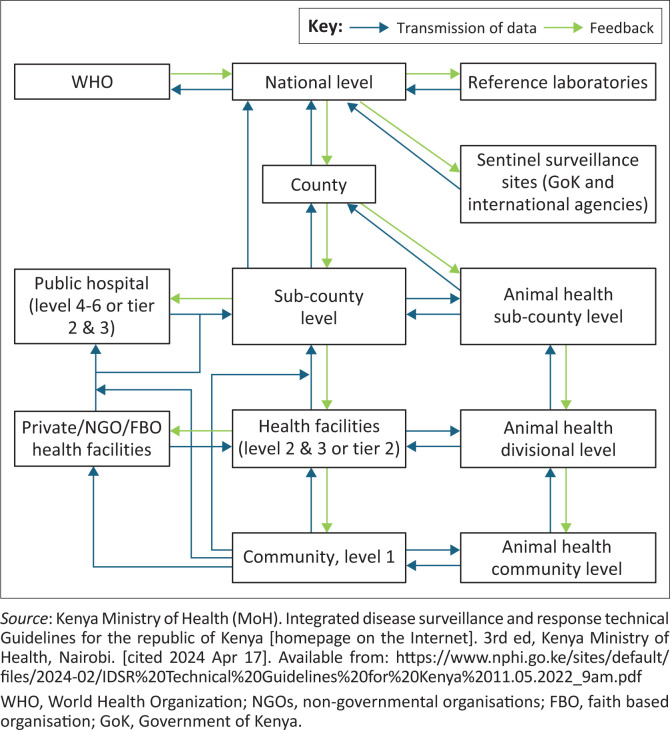
Integrated Disease Surveillance and Response (IDSR) Data Flow in Kenya.

During the 2014–2016 outbreak, there were no clear roles and responsibilities among stakeholders involved in the response. However, responders confirmed this was in place during the 2017–2019 outbreak. These included information flow (Figure 3)^14^, multi-sectoral coordination mechanism, laboratory and surveillance networks, cross-border and partner coordination and the linkage of laboratory systems with community and health facility surveillance.

#### Coordination and networks

The emergency operations centre was in place only during the 2017–2019 period and was activated for the first time which played a key role in coordinating the response at the national level. Partner coordination at the facilities stood at 60% with 40% indicating that there were no partner coordination mechanisms in their facilities during both periods. There were no available frameworks nor resources for partner engagements during both the periods:

‘The system consists of well-prepared human resources who are able to detect, notify and investigate an outbreak in a timely manner. However, other resources intended for coordination and investigation were limited.’ (KII H, sub-county surveillance officer, male)

In addition, responders underscored concerns about sustainability after the partners left:

‘I’m suggesting that they [*health partners*] should also be thinking about sustainability … At the end of their projects term, everything stalls.’ (FGD D, community health worker, female)

There was no multi-sectoral coordination meeting before this outbreak, and other sectors such as water and the environment were not optimally engaged:

‘It [*water department*] was not that strong compared to what we have right now. Right now, there is good coordination …’ (KII N, sub-county surveillance officer, male)

During both the periods, there were no cross-border coordination mechanisms established for cholera because of limited information of cholera outbreaks related to cross-border movements. Sixty per cent (*n* = 12/20) of facilities acknowledged existence of laboratory networks, which facilitated sample referrals, testing and interpretation of results.

#### Data management and information flow

During the 2017–2019 period, there was a clear guideline on how data were managed and reported from the community to the national level. However, this was not in place during the 2014–2016 period (Appendix 2). The information flow highlights that a suspected case of cholera is detected at the communities and facilities and then reported to the sub-county disease surveillance team which manages the case at the facility closest to the source with capacity.

#### System performance

**FIGURE 4:**
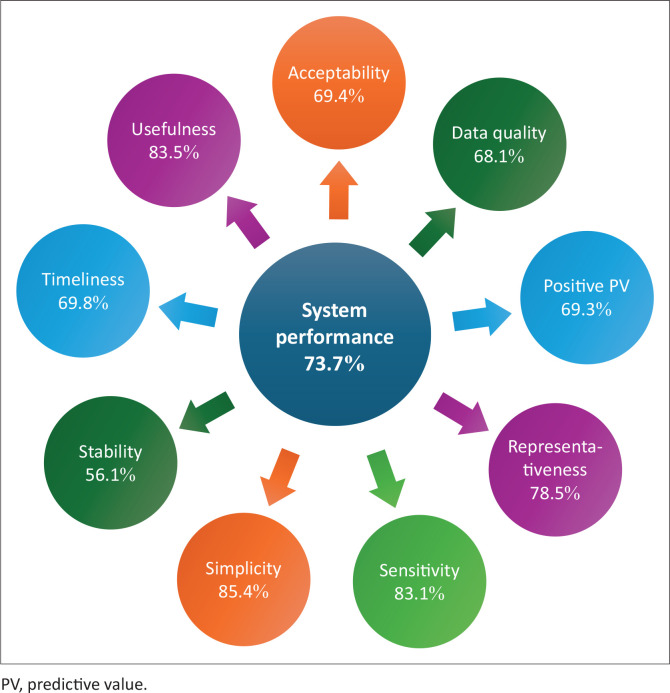
System attributes.

During the 2017–2019 period, the overall system performance was at 73.7%, with simplicity having the highest score at 85.4% and stability the least score of 56.1%. The system sensitivity, usefulness, acceptability, data quality, positive predictive value, timeliness and simplicity scores were 83.1%, 83.5%, 69.4%, 68.1%, 69.3%, 69.8% and 85.4%, respectively (Figure 4). It was widely agreed that the system’s ability to detect diseases was considerably good; however, the system was not able to respond to diseases on time:

‘The surveillance system works but timely resource availability hinders prompt and swift response.’ (KII D, sub-county surveillance officer, male)

## Discussion

Our findings demonstrate that there were indeed some improvements in the country’s capacity to detect, investigate and respond to the cholera outbreak comparatively.^5^ The rollout of the IDSR, surveillance and response guidelines, and SOPs and case definitions to the lower levels improved community engagement and prompt detection and reporting of cholera cases by community health workers. Even though the performance of the timeliness (for detection) indicator stood at 68.9%, this would have been better if the EBS system was fully operational.^15,16,17^ Other studies conducted in Kenya demonstrated that case detection in areas improved when SOPs, guidelines and case definitions are in place, and the community health workers are trained on the same as opposed to areas where these are missing.^18^ The availability of a cholera response plan just before 2017 also guided the response and facilitated coordination among stakeholders and partners^4^ Unlike in 2014, when there were no clear roles and responsibilities in reporting, the healthcare workers attested that they were clear with their reporting lines during the 2017–2019 outbreak. This finding aligns with another study from Nigeria which demonstrated that preparedness plans significantly improved early detection for Ebola and shortened the response time by the local authorities. The absence of such plans during the 2014–2016 period could have negatively reduced the effectiveness and speed in containing the outbreak.^19^ During both the periods, there was no harmonised mechanism for reporting at the community level, which further delayed standardising data across different communities and tracking cases in time. Some community health workers reported using a hard copy of the reporting form, while others reported collecting information through phone calls. Such a reporting mechanism has been proven to lead to incomplete and unharmonised reports and standards, delaying decision making and resource allocation during emergencies.^18^ These variations have now been addressed through the digital tool ‘m-Daura’ which is now serving as a platform for all community reports and EBS.^17^

During the 2017–2019 outbreak, there was an improvement in community engagement, and the community health workers could easily get relevant information from the community about cholera cases. This facilitated the penetration of the Ministry of Health (MoH) in hard-to-reach communities and facilitated early detection even in areas without proper hospitals, which aligns with the same results obtained from a similar study conducted in Haiti.^20^ Such workforce capacity could further be leveraged in the roll out of OCV in cholera hotspots. Even after a surge experienced in 2015, the Kenyan MoH established the Public Health Emergency Operating Centre (PHEOC) in 2016 only, and it was activated for the first time in 2017.^21,22^ Given the critical role PHEOC plays in coordinating outbreaks, this key milestone was a huge achievement in coordinating and controlling the outbreaks during 2017–2019 outbreak.^21^ A study conducted in Pakistan revealed the improvement in coordination and response to dengue when the PHEOC was established and activated.^23^ The decentralisation of the PHEOC could further improve outbreak coordination and response at sub-national levels while also lessening the burden on the national PHEOC during widespread outbreaks, as demonstrated by Nigeria and Uganda.^21,24^

Multisectoral collaboration was weak during both periods. The MoH, in collaboration with line ministries and partners, only convened the first national task force forum for the fight against cholera in July 2018. This was one of the setbacks in improving water hygiene and sanitation, which is a critical pillar in the control of the disease as demonstrated by previous publications.^25^

Laboratory systems and networks have been shown to be very instrumental in confirming cases during outbreaks for timely case management and response. However, the ability of countries to decentralise testing for advanced techniques such as culture and polymerase chain reaction (PCR) has always been a challenge. Even after this was highlighted as one of the challenges in the 2014–2016 period, the confirmatory tests were only conducted at the central level with a turnaround time of approximately 2 weeks. Such delays have been shown to sustain transmission chains in the communities by other studies.^26,27^ Rapid diagnostic test kits have been shown by other studies to bridge this gap by improving case detection, reducing the risk of misdiagnosis and inappropriate treatment at the community.^28^ However, while there was capacity at the sub-counties to perform RDTs, the kits were often limited in stock, leaving the communities to rely on suspected case definitions to detect them. A study conducted on the evaluations for sensitivity and cross-reactivity of Crystal VC (RDT test kits) used during the 2017–2019 outbreak, demonstrated a sensitivity of 97.5%, and specificity of 100% with no cross-reactivity; however, they were limited in stock.^29^ The Africa Centres for Disease Control and Prevention (CDC) has identified this as a critical gap on the African continent and is now supporting local manufacturing of laboratory test kits for priority diseases. The deployment of Field Epidemiology and Laboratory Training Program (FELTP) during the 2016–2017 played a key role in the control of the outbreak in urban cities. While there was significant support at the national level, the counties and sub-counties were not well-manned. Most of the staff deployed to the field at the sub-counties were mobilised by partners whose work ceased when the funding dwindled. Several countries also observed this scenario during sustained outbreaks such as coronavirus disease 2019 (COVID-19).^30^ In addition, the Kenyan health system relies mainly on voluntary commitment of community health assistants to detect and report health threats which has not been sustainable over the years.^31,32^ The formalisation of the engagement of community health workers as well as their formal integration into the healthcare system has shown to improve workforce development for emergency reponse.^33^

Training of healthcare workforce (laboratory, community health assistants, and volunteers and clinicians) was still considered one of the issues in both periods and it led hospital-acquired cholera infections in 2015.^5^ In 2017, most of the staff were seconded by partners and training was not properly conducted. The ripple effect is that, these system gaps translated to sub-optimal overall system performance revealed through the attributes. Of the 10 attributes (data quality, completeness, simplicity, timeliness, stability, usefulness, sensitivity, acceptability, representativeness, and positive predictive value) assessed, 3 (usefulness, sensitivity and simplicity) had acceptable performances of > 80%. The system stability scored lowest (56.1%) which was because of the uncertainty of staff contracts and unavailability of MoH computers for data capture. The breakdown of such devices usually leads to a disruption of workflow, given that it relies on the mercies of the staff for repairs.^34^ Health financing remains the driving force to ensure workforce sustainability and sustainable health financing. The total spending of health increased from $3476m accounting for 5.2% of the country’s gross domestic product (GDP)^35^ in 2015 to 8% in 2018,^36^ which is still below the 15% target of the Abuja declaration to which Kenya is a signatory. On the other hand, there was a decrease in budgetary allocations in health promotion and infectious disease prevention from 16 398m KES in 2015 to 7701m KES in 2015 and 6093m KES in 2015 to 5505m KES in 2017, which partly accounted for the poor water, sanitation, and hygiene (WASH) performance in counties and sub-counties without alternative funding source. The operationalisation of the Kenya National Public Health Institute will further strengthen the country’s health system governance and mobilise resources for disease prevention, detection and response. This would further strengthen key pillars such as workforce development, surveillance, laboratory, and emergency preparedness and response.

### Limitations

We noted several limitations in this study. Firstly, the sampling deployed was non-probability purposive sampling in selecting the counties and the facilities thus generalising the result to other countries might not be feasible. However, the qualitative aspect of the study provides deep insights that could support other countries with improving cholera preparedness, surveillance and response. Secondly, there were issues with recall bias given that some of the staff who were actively involved in both outbreaks had moved to other duties. Retrieving historic data was also challenging because the system depended on paper-based reporting during that period. We were able to support the findings with other data retrieved from hospital registers as well as national weekly reports which we could find on some websites. Lastly, we were not able to evaluate the effectiveness of the deployment of the RDTs during both periods which limited our understanding of the impact of its use between both the outbreaks.

## Conclusion

The Republic of Kenya has experienced an improvement in specific core capacities in cholera surveillance and response after 2014 to 2016 leading to decreased cases and deaths registered between 2017 and 2019. Improvements in areas such as the development and dissemination of guidelines and SOPs, defining information flow (including roles and responsibilities) across administrative levels and actors, activating the FELTP and establishment of a PHEOC were some major successes registered partner engagement and devolution that improved partner coordination. However, there remains sub-optimal performance in government financing and allocation of funds towards addressing public health emergencies. Establishing the National Public Health Institute will be an opportunity for such an agency to lead resource mobilisation and workforce development to support all the pillars of emergency response. Given the devolution of governance in Kenya, there is also an urgent need to explore the decentralisation of PHEOC and reference laboratories to reduce detection and confirmation time. The issue of shortage of RDT was a critical gap that can also be addressed via investment in local manufacturing. While there was no evidence for cross-border cholera transmission then, there is now ongoing cross-border transmission of cholera in the Horn of Africa and thus a need to strengthen the cross-border coordination to improve information sharing and joint response planning in border areas.

## Appendix 1

**TABLE 1-A1:** Regulatory documents, guidelines and standard operating procedures in Kenya critical to Cholera Surveillance and Response.

S/No.	Name	Purpose
**Regulations and Guidelines**		
1.	WHO/MSF Guidelines on Cholera Management	An effective response to cholera that involves engaging on several different fronts at the same time and as fast as possible to treat sick patients and to stop transmission within communities.
2.	International Health Regulation 2005	Kenya is a signatory to the 2005 IHR whereby it is stated that countries should strengthen surveillance and response capacity to be able to prevent, detect and control diseases promptly in a way that does not interfere with travel and trade.
3.	Integrated Disease surveillance and response strategy	Kenya has adopted the IDSR which is providing guidance in the detection and response to all priority diseases and conditions in the country. The strategy is also guiding all actors on their specific roles and responsibilities in disease surveillance and response in the country.
4.	*Public Health Act* (Cap 242)	This Act provides the protection of public health in Kenya and lays down rules relative to, among other things, food hygiene and protection of foodstuffs, the keeping of animals, protection of public water supplies, the prevention and destruction of mosquitos and the abatement of nuisances including nuisances arising.
5.	*The Eater Act* 2002	This Act provides for the regulation, management and development of water resources and water and sewerage services in line with the Constitution.
6.	Water Quality Regulations 2006	These regulations provide rules relative to the use and discharge of water for domestic, agricultural and industrial purposes, make provision for the protection of water resources from pollution and define water quality standards.
7.	*Food Drugs and Chemical Substances Act* (Cap 254)	It provides rules for the placing on the market of food and drugs for man and animal and of chemical substances; establishes the Public Health (Standards) Board; and makes otherwise provision for the control of the quality and safety of food, drugs and chemical substances to be placed on the market of Kenya.
8.	Cholera control guideline manual 2015	This guideline provides useful reference material for managers of health institutions, health workers involved in cholera treatment, and their supervisors. It is useful as an on-the-job manual, provides critical training areas and as a reference to both experienced and upcoming health professionals involved in the control and management of cholera epidemics.
**The standard operating procedures for cholera surveillance and response in Kenya typically include:**		
1.	Case definition	Clear criteria for identifying and classifying suspected and confirmed cases of cholera, including clinical symptoms such as watery diarrhoea and vomiting.
2.	Reporting mechanisms	Guidelines for healthcare facilities and public health authorities to promptly report suspected and confirmed cholera cases to the relevant authorities, such as the Ministry of Health or county health departments.
3.	Laboratory testing	Protocols for collecting, handling, and transporting stool samples for laboratory confirmation of cholera cases.
4.	Case management	Procedures for providing appropriate treatment and supportive care to cholera patients, including rehydration therapy and antibiotics.
5.	Contact tracing	Guidelines for identifying and monitoring individuals who may have been exposed to cholera cases to prevent further transmission.
6.	Environmental assessment	Protocols for investigating potential sources of cholera transmission, such as contaminated water sources or food, and implementing measures to mitigate the spread of the disease.
7.	Communication	Procedures for disseminating information about cholera outbreaks to healthcare providers, the public, and other relevant stakeholders, including risk communication messages and updates on the outbreak response.
8.	Coordination	Plans for coordinating cholera surveillance and response activities among different levels of the healthcare system, including local, regional, and national authorities, as well as with international partners and organisations.
9.	Training and capacity building	Strategies for training healthcare workers and other personnel involved in cholera surveillance and response on the relevant SOPs and protocols, as well as on infection prevention and control measures.
10.	Monitoring and evaluation	Mechanisms for monitoring the effectiveness of cholera surveillance and response activities, including regular reviews of surveillance data and outbreak investigations, and for making necessary adjustments to improve the response.

S/No., serial number; IDSR, Integrated Disease Surveillance and Response; IHR, International Health Regulations; MSF, Médecins Sans Frontières; WHO, World Health Organization; SOP, standard operating procedures.

## Appendix 2

**TABLE 1-A2:** Roles and responsibilities for various organisations in cholera prevention and control.

Institution/organisation	Function (roles and responsibilities)
**Government of Kenya**	
Ministry of Health (MoH)	MoH support the attainment of the health goals of the people of Kenya by implementing priority interventions in public health. The primary responsibilities of the MoH in cholera prevention and control include coordination of interventions (Division of Disease Surveillance and Response), laboratory confirmation, case investigation, formulation of policies and guidance, support for water, sanitation, and hygiene (WASH) activities (Department of Environmental Health and Division of Health Promotion), and regular training of staff at all levels of the health system.
Ministry of Education	Focusses primarily on school health programmes and can play an important role in strengthening cholera preparedness and response in national school health curricula. Regular treatment of school population to get rid of different parasitic infections. Conducting health education especially at the primary school level which is aimed at behaviour changes and adaptation of hygiene practices.
Ministry of Water and Sanitation	The Ministry’s primary functions include ensuring water regulation, increasing access to water, participating in emergency response, supporting the role of water companies in provision of safe water, and water quality monitoring. The link with cholera control is to ensure access of water at the local/community level or at the point of need, super-chlorination of water reservoirs and sewage management.
Ministry of Interior and Coordination of National Government	Involve the security apparatus from the inspector general of police to the village elders at the community level. The main role is to enforce and maintain law and order. The main mandate in cholera prevention and control is to enforce policies and regulations such as removal of illegal food vendors, illegal water connections and ensuring that all suspected cases are referred to a point of care.
Ministry of Environment and Forestry	To facilitate good governance in the protection, restoration, conservation, development and management of the environment and natural resources for equitable and sustainable development. Ensures protection of the water sources hence guaranteed water supply. Mitigates pollution of the environment including water bodies.
Kenya Medical Research Institute (KEMRI)	Conduct research in human health; to cooperate with other organisations and institutions of higher learning in training programmes and on matters relevant to research. Liaise with other institutions within and outside Kenya in carrying out research and related activities. Disseminate and translate research findings for evidence-based policy formulation and implementation.
Kenya Red Cross Society	Assisting the Kenyan Government to carry out humanitarian work in times of peace or conflict and during natural disasters, such as drought, famine, and floods. Their core activities include rapid response to outbreaks, distributing cholera kits to high-risk groups, and emergency preparedness. It also assists with human resource for health (workforce) and providing PPEs.
Kenyan Health Training Institutions	Many middle-level colleges, especially Kenya Medical Training College (KMTC) and the universities, now offer many health-related training courses and provide the human resources for health. The institutions’ health courses curriculum includes disease surveillance and cholera prevention and control.
The Kenya Field Epidemiology and Laboratory Training Program (FELTP)	FELTP is designed to train leaders in applied epidemiology and public health laboratory management, while providing epidemiologic services to national and sub-national health authorities in Kenya and beyond. Some of the core activities of the Kenya FELTP include conducting the Nairobi water quality survey, providing training in environmental testing, conducting outbreak investigations, and surveillance and supporting IDSR training.
**International organisations**	
World Health Organization (WHO)	WHO is the advisory and coordinating agent and authority for health within the United Nations’ system. It is responsible for providing leadership on health matters, advising on the priority international health agenda, setting norms and standards, articulating evidence-based policy options, shaping research agenda and eliciting generation of knowledge. It also provides technical support, monitoring and assessing health trends, and supports disease prevention and control programme implementation.
Center for Disease Control and Prevention (CDC)	CDC is a United States agency involved in health promotion, prevention, and preparedness and a global leader in public health. The CDC’s core activities in cholera prevention and control include: surveillance for disease; investigation of cholera outbreaks; risk assessments; development of training materials and courses for clinicians and community health workers; laboratory-based trainings and providing infection control in health facilities. Technical assistance to other sub-Saharan countries experiencing cholera, including countries neighbouring Kenya. A key technical partner (with funding from United States Agency for International Development [USAID]) in the design and implementation of the IDSR strategy.
United Nations Children’s Fund (UNICEF)	Works for children’s rights, their survival, development and protection. UNICEF core functions include sponsoring health, nutrition and WASH programming including support for health worker training. UNICEF’s efforts in WASH including its support for the development of the sanitation strategy, have greatly contributed to cholera prevention and control in Kenya.
United States Agency for International Development (USAID/Kenya)	USAID Kenya’s Maternal and child health program focusses on prevention and management of diarrhoeal diseases in children under five through creating awareness on prevention among communities, promoting appropriate treatment among service providers and coordination at national level. In addition, USAID is working to strengthen Kenya’s health system overall by improving policy, logistics, health worker effectiveness, and monitoring and evaluation.
Global Task Force on Cholera Control (GTFCC)	A multi-sectoral partner that serves as an effective platform to support countries in the implementation of Ending Cholera: A Global Roadmap to 2030, which targets a 90% reduction in cholera deaths and cholera elimination in 20 countries.
**Local health implementing partners and NGOs**	
Médecins Sans Frontières (MSF)	MSF is an international organisation for medical humanitarian aid with a special emphasis on medical emergency response. In relation to cholera, MSF is involved in quick response and has good experience managing cases under challenging areas with poor health infrastructure.
The Safe Water and AIDS Project (SWAP)	SWAP is a non-governmental organisation based in Western Kenya (former Nyanza, Westerns and parts of Rift-valley province) that engages HIV support and self-help groups to promote and sell water treatment and other health products as an income-generating activity that also benefits the wider community. Core activities with respect to cholera include a rapid outbreak response element. The rapid response teams have responded to over a dozen cholera outbreaks in Kenya.

IDSR, Integrated Disease Surveillance and Response; NGOs, non-governmental organisations; PPE, personal protective equipment.
